# The complete chloroplast genome of *Changnienia amoena* S. S. Chien (Orchidaceae) and its phylogenetic implication

**DOI:** 10.1080/23802359.2019.1699464

**Published:** 2019-12-12

**Authors:** Xiangui Yi, Mingzhi Li, Lin Chen, Xianrong Wang

**Affiliations:** a Co-Innovation Center for Sustainable Forestry in Southern China, Nanjing Forestry University, Nanjing Shi, China;; b College of Biology and Environment, Nanjing Forestry University, Nanjing Shi, China;; c Key Laboratory of State Forestry Administration on Subtropical Forest Biodiversity Conservation, Nanjing Forestry University, Nanjing Shi, China;; d Biodata Biotechnologies Co. Ltd, Hefei, China

**Keywords:** *Changnienia amoena*, complete chloroplast genome, phylogenetic analysis, Orchidaceae

## Abstract

*Changnienia amoena* S. S. Chien is mainly distributed in the mid-subtropical hilly areas of central and eastern China at an altitude of 200–1700 m. It has important academic significance in the orchid phylogeny, and is an excellent wild flower and precious medicinal plant. The species was once abundant but has become rare and endangered in recent years and has been identified as Endangered (EN) under criteria A2c (The IUCN Red List and Threatened Specis) because of the habitat fragmentation and unduly commercial collections. In this study, the complete chloroplast (cp) genome sequence of *C. amoena* was determined using next-generation sequencing. The entire cp genome was determined to be 156,818 bp in length. It contained large single-copy (LSC) and small single-copy (SSC) regions of 84,847 and 18,141bp, respectively, which were separated by a pair of 26,915 bp inverted repeat (IR) regions. The genome contained 133 genes, including 87 protein-coding genes, 38 tRNA genes, and eight rRNA genes.The overall GC content of the genome is 37.1%. A phylogenetic tree reconstructed by 48 chloroplast single-copy coding gene reveals that *C. amoena* is closely related with *Calypso bulbosa*.

*Changnienia amoena* belongs to the Orchidaceae family Calypsoinae and is a unique species of genus *Changnienia* in China (Dressler 1993). It is a monotypic species and endemic to China (IUCN 2019), has important academic significance in the orchid phylogeny, and was listed on the Chinese Red Book in 1992 (Fu 1992). The species was once abundant but has become rare and endangered in recent years because of the habitat fragmentation and unduly commercial collections. So, it is necessary to develop genomic resources for *C. amoena* to provide basic intragenic information for further study on phylogeny and bio-conservation in genus *Changnienia.*

The total genomic DNA was extracted from the fresh leaves of *C. amoena* (32.131738 N 119. 089981E, altitude 230 m) using the DNeasy Plant Mini Kit (Qiagen, Valencia, CA, USA). The voucher specimen was deposited at the Herbarium of Nanjing Forestry University (YXG17041501). The whole genome sequencing was conducted by Hefei Biodata Biotechnologies Inc. (Hefei, China) on the Illumina Hiseq 4000 Sequencing System (Illumina, Hayward, CA). The filtered sequences were assembled using the programme SPAdes assembler 3.10.0 (Bankevich et al. 2012). Annotation was performed using the DOGMA (http://dogma.ccbb.utexas.edu/) .

The plastome of *C. amoena* was determined which comprised double-stranded, circular DNA of 156,818 bp containing two inverted repeat (IR) regions of 26,915 bp each, separated by large single-copy (LSC) and small single-copy (SSC) regions of 84,847 and 18,141 bp, respectively (NCBI acc. no. MN047293). The genome contained 133 genes, including 87 protein-coding genes, 38 tRNA genes, and 8 rRNA genes. The eight protein-coding genes, eight tRNA genes, and four rRNA genes were duplicated in IR region. Nineteen genes contained two exons and four genes (clpP and ycf3, and two rps12) contained three exons. The overall GC content of *C. amoena* cp genome is 37.1% and the corresponding values in LSC, SSC, and IR regions are 34.8, 29.8, and 43.2%, respectively.

To investigate its taxonomic status, a maximum likelihood (ML) was reconstructed based on 48 chloroplast single-copy coding gene from 43 plants in Orchidaceae (using the outgroup *Phyllostachys sulphurea*) by FastTree version 2.1.10 (Figure 1) (Price et al. 2010). The ML phylogenetic tree shows that *C. amoena* is most related with *Calypso bulbosa* in Orchidaceae, with bootstrap support values of 100%.

**Figure 1.**
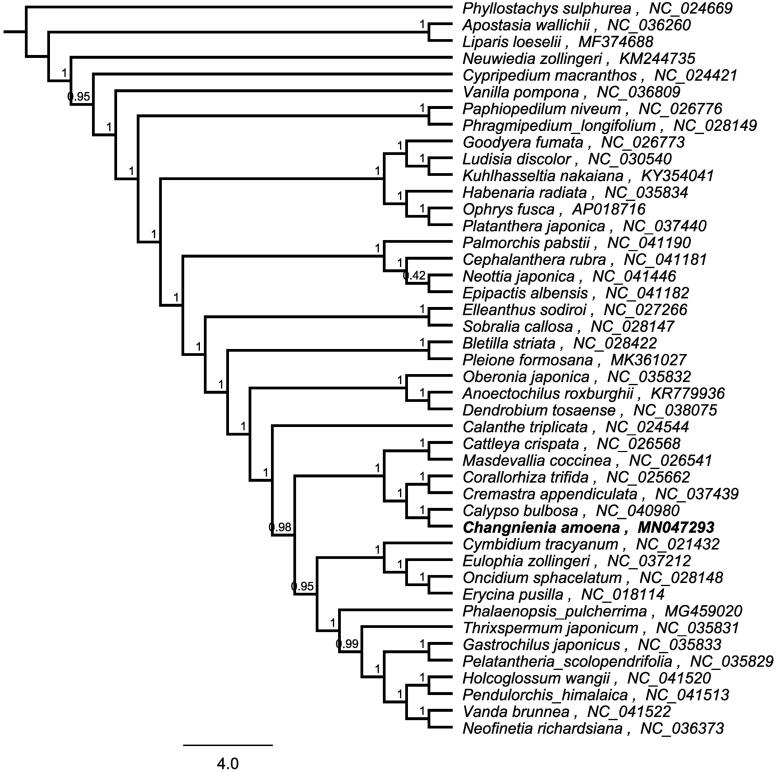
Maximum-likelihood phylogenetic tree for *Changnienia amoena* based on 48 chloroplast single copy coding gene from 43 plants in *Orchidaceae*. *Phyllostachys sulphurea* were used as outgroup and the support values are shown at the branches.
